# Surgery

**Published:** 1895-11

**Authors:** 


### SURGERY.

EDITED BY FLOYD W. MCRAE, M.D.

### THE DANGER SIGNAL OF THE CHLOROFORMIST.

The opinion of most of the expert anesthetists in contradiction to the suggestions of the Hyderabad Chloroform Commission, is that the respiration alone is an imperfect guide to the condition of a patient under chloroform, and the pupil, pulse, and the patient, as a whole, should be watched.

It certainly seems probable that if we watch the respiration alone we are in danger of pushing the chloroform to the point of respiratory narcosis; and since this will come on gradually, we may not recognize the condition until the patient is in a state of extreme danger.

It may be true that the patient can always be brought around by artificial respiration; this, involving as it does, the stopping of the operation in many cases, is a most inconvenient and alarming complication, and should never be allowed to occur.

Any interference with the respiratory center by chloroform, however slight, is a sign of overdosing. Again if respiration alone be watched, how is the beginner to distinguish between shallow and irregular respiration of the reflex inhibition, which so often precedes vomiting, and the insidious onset of respiratory narcosis? If chloroform be pushed to the point of affecting the pulse, if this be possible, a dangerous overdose has been given. The heart, too, is liable to reflex inhibitions, and often becomes irregular and depressed during vomiting, and also during the violent irritation of the sympathetic system met with in abdominal operations; in either case, quite independently of the anesthetic. Therefore, as an indication of the degree of the chloroform narcosis, the pulse is unreliable.

We require some indication which shall tell us when the cerebrum is completely narcotized, and shall also warn us when we are in danger of affecting the respiratory center. This indication is found in the pupil. The third nerve center which governs it is the first of the automatic centers of which we have cognizance; it is not a vital center, like the respiratory, and its narcosis is not, in itself, followed by dangerous complications. The pupil, which is the visible sign of the condition of this center, is the danger signal for which we are looking. The pupil has a regular cycle as the patient goes under. It is first dilated and active, it then becomes contracted, and lastly it becomes dilated and fixed. The first state is a sign of imperfect narcosis, the second of complete and safe narcosis, and the third of danger of imminent narcosis of the respiratory center. The cause of this cycle is as follows: Imperfect narcosis, going under or coming round, the pupil is dilated because impulses, mental, sensory or sympathetic, affect the half-narcotized cerebrum, and cause reflex inhibition of the third nerve center; and active because the center itself has not been reached by the anesthetic. A similar dilation is produced under ordinary conditions by fright, pain, or a blow on the abdomen. As narcosis deepens, the pupil contracts because the cerebrum is completely under, all cerebral reflexes are barred, and the third nerve center is consequently unimpeded in its action. A similar state is seen in deep sleep. If the narcosis be pushed further, the pupil will slowly dilate and become less active to light till it is widely dilated and fixed, because the narcosis has now reached the center itself, and has gradually overtaken it; consequently nervous control has ceased and the pupil has dilated; at the same time the light reflex has been abolished. A similiar condition of pupil is seen in general cerebral compression. This fixed dilatation indicates great danger, for respiratory narcosis is imminent; indeed, under no circumstances should narcosis be pushed to the extent of full dilatation of the pupil. Thus the golden mean of safety is indicated by a contracted pupil, any material dilatation means "look out." The patient is either coming round and developing reflexes, or going too far toward respiratory narcosis. It is easy to distinguish between commencing reflex dilatation and early narcosis dilatation. In the first, the pupil is active and other reflexes—shallow respiration, vomiting, or movements—will follow; in the second, the patient is stertorous, the pupils sluggish and the eyeballs fixed. In the first, the indication is for more chloroform; in the second, for the suspension of the drug till contraction recurs in consequence of the recovery of the third nerve center. If the pupil be read in this way all interference with respiration of the heart can be avoided.

For all ordinary operations, a contracted pupil should be maintained, but in abdominal surgery it is sometimes necessary to combat the violent sympathetic irritation by pushing the chloroform till there is slight narcosis dilatation of the pupil. Beyond this, it is useless, as well as dangerous, to go. Any further abdominal rigidity is due either to inflammatory fibrosis, or to the development of abnormal reflex links between the sympathetic and spinal nerves in highly neurotic subjects. In either case the condition is beyond the control of anesthesia.

The time during which the closest attention should be paid to the respiration is while the patient is going under. At this time he is liable, intentionally or from too strong a vapor, to hold his breath. The respiratory center is thus debilitated from lack of oxygen; then when the necessity for breathing overcomes all other impulses, a gasping inspiration is taken, the center is flooded with chloroform, and cannot resist it, the pupil dilates, and death supervenes. Whether or not the heart is affected is undetermined, the point being to avoid the occurrence in any case. This can be done by encouraging the patient to breathe regularly, and if he holds his breath, by seeing that only a small dose of chloroform is accessible.

In children it is better to use a Junker's inhaler, so that whether they scream or hold their breath, only a limited amount of vapor can be taken in; an overdose is thus avoided. In these cases it is my practice to give chloroform till contraction occurs with slight stertor, and then to suspend the administration until some slight reflex is seen, then to give a little more, and so on. I do not think that the pupil is quite so reliable as in the adult, as sudden overdosing with fixed dilatation seems sometimes to occur. Possibly the pupil reflex is imperfectly developed; this I am sure is the case with the corneal reflex, which is quite unreliable as a sign of narcosis in children. Slight stertor is the reliable indication.—*Arthur H. Ward, M.D., in the Lancet.*

---

### ACETANILID AS A SURGICAL DRESSING.

At a recent meeting of the Philadelphia County Medical Society Dr. Thomas S. K. Morton read a report of the use of acetanilid in surgery, based upon his observations in an extended hospital and private practice. The results were so exceedingly favorable that he recommended its general adoption. In all operation on wounds, including amputations, used as a dusting powder, it promoted healing without suppuration. Its effect was to thoroughly dry the wound so that the usual drainage tube was not necessary. In cases of scalds and burns it dries and soothes the affected surface, allowing healing to take place rapidly. As a dressing to chronic sores and ulcers it proved superior to other dressings. But two cases of constitutional effects were observed, one in his own practice and one reported by a surgeon in California; and, in both, the powder was applied over a large area in cases of extensive burns. Neither case was fatal.

Acetanilid is very cheap, being at present only forty cents per pound. It is colorless and non-odorous. It is soluble in five parts of alcohol, and in four-tenths parts of boiling alcohol; in 194 parts of water at 59° F., and in eighteen parts of boiling water, in eighteen parts of ether, in equal parts of chloroform, and in the various petroleum oils in the proportion of forty grains to the ounce. In powder form it may be mixed with pulverized starch, boracic acid, or, if an alkaline powder be desired, bicarbonate of sodium. The best preparations are as follows:

For dusting powder, pure acetanilid, or one ounce of acetanilid to two to four ounces of starch, freely triturated; for a solution, one drachm of acetanilid dissolved in five drachms of alcohol, to which add about eight ounces of boiling water and let cool; for an oily solution, forty grains of acetanilid to one ounce of liquid petrolatum; for an ointment, forty grains of acetanilid to one ounce of petrolatum; for a collodion, forty grains of acetanilid to one ounce of collodion. These proportions will be modified by the practitioner to suit particular cases as his experience advances.—*Indiana Medical Journal.*

---

### THE ANTISEPTIC TREATMENT OF BURNS.

An eminent French surgeon recently concluded an article with the above heading in the following words:

1. Fresh, superficial burns, as well as deep ones, can heal under antiseptic treatment without the production of pus.
2. If pus is produced, the wound is disinfected, and the course remains the same as if non-infected. But if the pus is of long standing and the wound begins to granulate, then disinfection is not possible.
3. To disinfect widespread burns an anesthetic will often be necessary, and to this end chloroform is best suited.
4. If the wound is non-purulent, the unnecessary use of an antiseptic hinders the healing process.
5. Antisepsis is the best analgesic.
6. Burns heal rapidly under the antiseptic treatment. Burns of the second degree require eight days; of the third degree, from two to three weeks.
7. Burns of the second and third degree heal without trace remaining; of the fourth degree, cause a scar, which does not retract, while this will be smoother the less the amount of pus.—*Charlotte Med. Journ.*

